# Role of GLP-1 receptor agonists in the prevention and treatment of obesity-related cancer

**DOI:** 10.1007/s11739-026-04458-z

**Published:** 2026-07-07

**Authors:** Giovanna Muscogiuri, Guendalina Del Vecchio, Luca Colangeli, Valeria Guglielmi, Paolo Sbraccia, Annamaria Colao

**Affiliations:** 1 Unità Di Endocrinologia, Diabetologia E Andrologia, Dipartimento Di Medicina Clinica E Chirurgia, Università Degli Studi Di Napoli Federico II, Via Sergio Pansini 5, 80131 Naples, Italy; 2 Cattedra Unesco, Educazione Alla Salute E Allo Sviluppo Sostenibile, University Federico II, 80131 Naples, Italy; 3 Department of Medicine, Division of Endocrinology, University of Arizona, Tucson, AZ USA; 4 Department of Systems Medicine, University of Rome Tor Vergata, Viale Montpellier 1, 00133 Rome, Italy; 5 Internal Medicine Unit, Obesity Center, University Hospital Policlinico Tor Vergata, Rome, Italy

**Keywords:** GLP-1 RAs, Semaglutide, Obesity-related cancer, Cancer, Prevention, Adjuvant treatment, Safety

## Abstract

Glucagon-like peptide-1 receptor agonists (GLP-1 RAs) have become pivotal in treating type 2 diabetes and obesity due to their ability to improve glycemic control and promote weight loss. Beyond their metabolic benefits, emerging evidence suggests that GLP-1 RAs may exert anticancer effects by modulating critical biological pathways involved in tumorigenesis, including insulin signaling, inflammation, and cellular proliferation. Current evidence derived from observational and secondary analyses suggests potential protective effects of GLP-1 RAs against several cancers—including prostate, breast, pancreatic, gynecological, glioma, and oral squamous cell cancer through both weight-dependent and independent mechanisms, but the absence of large-scale randomized controlled trials (RCTs) with cancer-specific endpoints remains a critical limitation. Moreover, safety concerns remain a major challenge. Although rodent studies raised concerns regarding medullary thyroid carcinoma, human data have not substantiated a significant risk. Similarly, initial signals of pancreatitis and pancreatic cancer risk have been largely attenuated by subsequent rigorous analyses. Common side effects, such as gastrointestinal discomfort, are usually manageable and transient, but rare adverse events warrant vigilance, particularly in vulnerable patient populations. GLP-1 RAs show emerging signals for cancer prevention and treatment, but their therapeutic application in oncology should proceed cautiously. Future research must prioritize well-designed, adequately powered cancer-focused RCTs that evaluate both efficacy and safety over extended periods.

## Introduction

Glucagon-like peptide-1 receptor agonists (GLP-1 RAs) have emerged as transformative agents in the management of type 2 diabetes (T2D) and obesity, primarily due to their efficacy in improving glycemic control and inducing weight loss [1]. Beyond their metabolic effects, recent research suggests that GLP-1 RAs may play an important role in cancer prevention and therapy, given their ability to modulate key biological pathways implicated in tumorigenesis, such as insulin signaling, inflammation, and cellular proliferation [2, 3]. These potential anticancer properties have led to growing interest in repurposing GLP-1 RAs for oncology applications [2, 3].

Despite promising preclinical and observational data, concerns regarding the safety of GLP-1 RAs persist [4]. Notably, early animal studies indicated a risk of medullary thyroid carcinoma (MTC) in rodents treated with GLP-1 RAs, raising alarms about a possible carcinogenic effect [5, 6]. However, this finding has not been convincingly demonstrated in humans, although regulatory agencies maintain warnings for GLP-1 RAs use in patients with a history of MTC or multiple endocrine neoplasia syndrome type 2 (MEN2) [5, 6].

Another significant concern involves the potential risk of pancreatitis and pancreatic cancer [7]. Initial reports and pharmacovigilance data suggested an association between GLP-1 RA use and pancreatitis, which could theoretically increase pancreatic cancer risk. Nevertheless, extensive meta-analyses and cardiovascular outcome trials have largely refuted this association, indicating that any increased risk, if present, is likely minimal [7]. Other adverse effects include common gastrointestinal symptoms such as nausea and vomiting, which are generally mild and transient [8]. Rarely, hypersensitivity reactions, gallbladder disease, and increased heart rate have also been reported [9], but their implications in cancer patients remain unclear.

A critical limitation in the current evidence base is the paucity of large-scale randomized controlled trials (RCTs) specifically designed to evaluate cancer incidence, progression, or mortality as primary endpoints [2, 3]. Much of the available data are derived from secondary analyses of metabolic or cardiovascular trials, retrospective observational studies, and preclinical models. This limits the strength and applicability of the findings related to cancer outcomes. Additionally, variability in study populations, cancer types, and treatment regimens complicates the interpretation of results. Many studies have relatively short follow-up periods, insufficient to capture long-term cancer outcomes, which may take years to manifest. The use of observational data introduces potential confounding factors and biases, such as selection bias and confounding by indication, which challenge the drawing of definitive conclusions [2, 3].

Given these safety concerns and methodological limitations, there is a clear need for comprehensive and rigorous investigation into the oncological safety and efficacy of GLP-1 RAs. This review aims to critically assess current evidence regarding the safety profile of GLP-1 RAs, highlight the limitations of existing studies, and identify gaps requiring further research to establish the role of GLP-1 RAs in cancer prevention and treatment.

To provide a comprehensive overview of the role of GLP-1 RAs in obesity-related cancer, a literature search was conducted across PubMed, Scopus, and Web of Science databases up to December 2025. The search strategy utilized keywords including ‘GLP-1 receptor agonists’, ‘semaglutide’, ‘liraglutide’, ‘tirzepatide’, ‘obesity’, and ‘cancer’ or specific malignancies (e.g., ‘colorectal’, ‘breast’, ‘pancreatic’). We prioritized peer-reviewed original research, large-scale observational studies, and recent meta-analyses, focusing on English-language publications that provided significant mechanistic insights or clinical evidence regarding the safety and efficacy of these therapies.

## Weight-related pathophysiological mechanisms

**Fig. 1:**
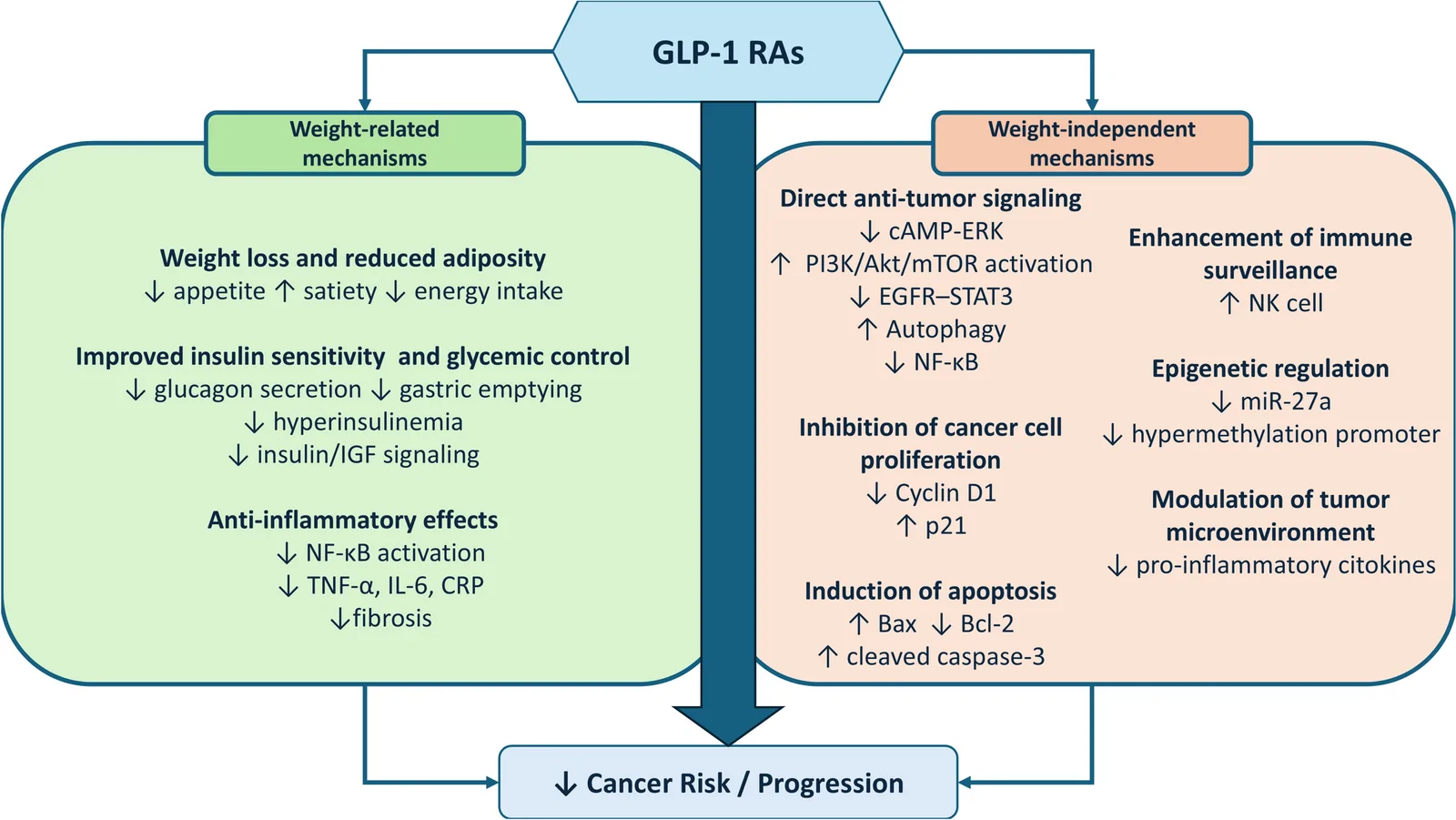
Weight-related and weight-independent mechanisms. *CRP* C-reactive protein, *GLP-1 RA*s GLP- 1 receptor agonists, *IGF* insulin-like growth factor, *IL-6* interleukin-6, *miR-27a* microRNA-27a, *NK* natural killer, *TNF-α* tumor necrosis factor α

GLP-1 RAs have emerged as powerful tools in managing obesity and T2D, and mounting evidence suggests they may also reduce cancer risk and hamper tumor progression through multiple metabolic and non-metabolic pathways [10] (Fig. 1).

First, GLP-1 RAs improve insulin sensitivity and glycemic control [11]—two critical factors implicated in cancer risk [12]. Hyperinsulinemia and insulin resistance are known to drive oncogenesis by activating insulin receptor and insulin-like growth factor (IGF) signaling pathways, which promote cellular proliferation, migration, invasion, and suppression of apoptosis [12]. GLP-1 RAs exert anti-hyperglycemic effects by inhibiting glucagon secretion, slowing gastric emptying, enhancing insulin sensitivity, and stimulating insulin secretion [11]. This improvement in metabolic homeostasis likely reduces insulin mediated pro-tumorigenic signaling.

Second, by inducing weight loss and improving lipid metabolism, GLP-1 RAs tackle obesity—a well-recognized, independent risk factor for various cancers [13]. Obesity fuels chronic inflammation, adipose tissue remodeling, and dysregulated adipokine secretion—e.g., elevated leptin and reduced adiponectin—fostering tumor cell proliferation, invasion, and metastasis [13]. Liraglutide and other GLP-1 RAs promote satiety, suppress appetite, and reduce energy intake, resulting in substantial weight loss [14]. Moreover, they may enhance energy expenditure via browning of white adipose tissue, increased lipolysis, and thermogenic activation mediated by interleukin-6 (IL-6) signaling [14].

Third, GLP-1 RAs exert anti-inflammatory effects in adipose tissue and systemically [15, 16]. They suppress NF-κB signaling and reduce production of pro-inflammatory cytokines such as tumor necrosis factor- α (TNF-α), IL-6, and C-reactive protein (CRP), independently of weight change [15, 16]. In models of liver inflammation and nonalcoholic steatohepatitis (NASH), GLP-1 RAs reduce hepatic fat, inflammation, and fibrosis, thereby lowering the risk of progression to hepatocellular carcinoma (HCC) [17]. These anti-inflammatory pathways further disrupt the tumor promoting microenvironment associated with obesity.

By simultaneously improving insulin resistance, reducing adiposity, and alleviating inflammation, GLP-1 RAs mitigate several obesity-driven cancer-promoting processes. Yet, their anti-cancer potential cannot be fully explained by these metabolic benefits alone, highlighting the importance of weight-independent mechanisms that act directly on tumor biology.

## Weight-independent pathophysiological mechanisms

In addition to their systemic metabolic effects, GLP-1 RAs exhibit weight-independent mechanisms that directly influence tumor biology, including effects on proliferation, apoptosis, oncogenic signaling, and immune surveillance (Fig. 1).

### Inhibition of cancer cell proliferation

Multiple studies have documented that GLP-1 RAs can inhibit the proliferation of various cancer cell lines [18, 19]. Mechanistic studies show that GLP-1 RAs can bind the GLP-1 receptor (GLP-1 R) and initiate signaling cascades that inhibit proliferation, induce apoptosis, and block tumor cell migration and invasion in various cancer cell lines. For example, liraglutide and exenatide suppress growth in breast, pancreatic, and prostate cancer models [20, 21]. This antiproliferative effect appears to result from GLP-1 R activation, which initiates downstream signaling cascades that reduce cell cycle progression. In breast cancer cells, GLP-1 RAs downregulate cyclin D1 and upregulate cell cycle inhibitors such as p21, effectively halting cell division [22]. Similarly, pancreatic cancer cell lines exposed to GLP-1 RAs show decreased proliferation markers and impaired tumorigenicity in xenograft models [23].

### Induction of apoptosis

Induction of programmed cell death is a vital anticancer mechanism. GLP-1 RAs have been shown to promote apoptosis in diverse cancer types through mitochondrial and death receptor pathways [19]. In prostate cancer cells, liraglutide increases the expression of pro-apoptotic proteins such as Bax and cleaved caspase-3, while reducing anti-apoptotic Bcl-2 levels [24]. Similar pro-apoptotic effects were reported in glioma and colorectal cancer models, where GLP-1 RAs triggered mitochondrial dysfunction and caspase activation [24]. This apoptosis induction is partly mediated by modulation of intracellular signaling pathways critical for cell survival. Emerging evidence suggests that GLP-1 RAs may also exert anticancer effects by modulating ferroptosis, a specialized form of regulated cell death. It has been demonstrated that these agents can influence iron metabolism and lipid peroxidation pathways, potentially sensitizing certain cancer cells to ferroptotic stimuli or protecting healthy tissues from oxidative damage [25].

### Modulation of key signaling pathways

The ability of GLP-1 RAs to regulate oncogenic signaling has been extensively documented. They influence pathways such as cAMP-ERK inhibition, suppression of EGFR–STAT3, activation of autophagy, and downregulation of NF-κB [20].

In particular, activation of AMPK leads to energy homeostasis restoration and inhibits anabolic processes necessary for tumor growth [26]. GLP-1 RAs stimulate AMPK phosphorylation in cancer cells, which suppresses mTOR activity, thereby reducing protein synthesis and cell proliferation [27]. In addition, GLP-1 RAs inhibit the phosphatidylinositol 3-kinase/protein kinase B/mammalian target of rapamycin (PI3K/Akt/mTOR) pathway, a common driver of tumor progression and resistance to therapy. By attenuating this signaling cascade, GLP-1 RAs hinder cancer cell survival and promote apoptosis. For example, liraglutide treatment decreased phosphorylated Akt levels in pancreatic cancer cells, enhancing sensitivity to chemotherapy [28].

### Enhancement of immune surveillance and epigenetic modulation

GLP-1 RAs also modulate the tumor microenvironment through immune and epigenetic mechanisms [29]. Specifically, these agents enhance natural killer (NK) cell function, an important component of the innate immune system responsible for targeting and eliminating malignant cells. GLP-1 RAs have been shown to increase NK cell cytotoxicity and cytokine production, which may contribute to tumor immune surveillance [30]. This immune activation is crucial for controlling micrometastases and preventing cancer recurrence [31]. Additionally, epigenetic and microRNA (miRNA)-mediated regulation has been reported. In breast cancer, GLP-1 RAs inhibit miR-27a, activate AMPKα2, reduce promoter hypermethylation of tumor suppressor genes (e.g., ESR1, CDH1, ADAM33), and restore epithelial phenotype, thereby suppressing invasion and therapy resistance [27].

## Clinical and epidemiological evidence

Chronic low-grade systemic inflammation represents a central biological link between obesity and cancer development. Excess adiposity is characterized by adipocyte hypertrophy, hypoxia, and macrophage infiltration within visceral adipose tissue, leading to a phenotypic shift toward pro-inflammatory M1 macrophages. This process results in increased circulating levels of TNF-α, IL-6 and CRP, together with activation of NF-κB and JAK/STAT3 signaling pathways. Persistent activation of these inflammatory cascades promotes genomic instability, angiogenesis, tumor cell proliferation, immune evasion, and metastatic potential [15, 16]. GLP-1 RAs have consistently demonstrated systemic anti-inflammatory effects in patients with obesity and T2D [32]. Meta-analyses of randomized controlled trials report significant reductions in circulating CRP, IL-6, and TNF-α levels following GLP-1 RA therapy [15, 33]. Notably, these effects appear to be only partially attributable to weight loss, suggesting direct immunomodulatory properties that may underlie the therapeutic benefits observed in inflammatory dermatologic and rheumatologic conditions, including psoriasis [34].

While preclinical and mechanistic studies provide important insights into the antitumor actions of GLP-1 RAs, the ultimate test of their relevance lies in clinical and population-level data. Over the past few years, a growing body of real-world evidence, retrospective cohort analyses, and meta-analyses has begun to shed light on the potential cancer-protective role of GLP-1 RAs.

Large population-based cohort studies have provided interesting evidence suggesting that GLP-1 RA therapy may be associated with reduced incidence of obesity-related cancers [2]. A landmark retrospective cohort study analyzing over 86,000 subjects demonstrated that GLP-1 RA users had a significantly lower overall cancer incidence compared with non-users, with hazard ratios indicating risk reductions particularly for endometrial, ovarian, and meningioma cancers. These findings are consistent across multiple independent cohorts [2].

Similarly, a nationwide observational study involving over one million patients with obesity reported differential cancer risk modification by various GLP-1 RAs [35]. Notably, semaglutide was associated with decreased gastrointestinal cancer incidence, while liraglutide showed some associations with thyroid and respiratory cancer risks, indicating possible drug-specific effects [35].

Meta-analyses aggregating data from multiple studies highlight the protective effects of GLP-1 RAs against hepatocellular carcinoma, a common obesity-associated malignancy [36]. One systematic review encompassing eight studies showed a significant 59% reduction in HCC risk in GLP-1 RA-treated patients compared to insulin-treated or untreated controls [36]. Another meta-analysis reported consistent decreases in liver decompensation and HCC occurrence among GLP-1 RA users [36].

Evidence on tirzepatide, a dual GIP/GLP-1 receptor agonist, remains limited due to its recent clinical introduction and the relatively short follow-up of available studies. A subgroup analysis of very recent meta-analysis encompassing 25 randomized trials with 40,731 participants suggested that co-agonists (tirzepatide, cotadutide, and cagrilintide) were associated with a significant reduction in overall obesity-related cancer risk, an effect that appeared to be partially mediated by weight loss magnitude [37].

While long-term cancer incidence data from RCTs remain limited due to relatively short follow-up durations, pooled analyses of cardiovascular outcome trials have begun to reveal favorable cancer-related trends. A meta-analysis of RCTs comparing GLP-1 RAs to placebo or other antidiabetics found no increase in overall cancer risk, with some evidence pointing to reduced uterine cancer incidence among patients treated with GLP-1 RAs [38].

Real-world analyses from large health registries corroborate these findings. A U.S. cohort study involving 1.6 million patients with T2D revealed significantly lower risks of ten obesity-related cancers (including colorectal, endometrial, ovarian, hepatic, pancreatic, and meningioma) among GLP-1 RA users compared to insulin users [3]. Moreover, a comparative study in Israel demonstrated nearly 50% lower obesity-related cancer risk among GLP-1 RA users versus patients undergoing bariatric surgery, underscoring possible weight-independent anti-cancer mechanisms [39].

A further point that warrants clarification is whether the potential antitumor effects of GLP-1 RAs are comparable between patients with T2D and individuals with obesity without diabetes. In patients with T2D, anticancer effects may be partially mediated by improved glycemic control and reductions in hyperinsulinemia, both recognized contributors to cancer risk [12]. In contrast, in individuals with obesity without T2D, weight loss, modulation of adipokines and attenuation of chronic low-grade inflammation are likely to represent the predominant mechanisms [15, 16]. It should be acknowledged that most large-scale clinical and observational data currently derive from T2D populations; therefore, extrapolation to individuals with obesity without diabetes warrants caution. However, given the central role of visceral adiposity in the pathogenesis of T2D, a meaningful proportion of the observed cancer risk reduction in T2D may be driven by adiposity-related mechanisms. In addition, emerging evidence suggests that GLP-1 RAs exert weight-independent anti-inflammatory and immunomodulatory effects [34], which may contribute to potential anticancer properties across both populations.

Nonetheless, observational studies cannot definitively establish causality due to confounding factors, such as healthier patient profiles and differences in cancer screening. Moreover, meta-analyses often suffer from heterogeneity and low event rates. Therefore, long-term RCTs specifically designed to assess cancer endpoints in both T2D and non-diabetic obesity populations are critical to confirm the protective role of GLP-1 RAs against cancer.

## Cancer types associated with GLP-1 receptor agonist effects

Emerging evidence suggests that the impact of GLP-1 RAs on cancer risk and progression may vary depending on tumor type, reflecting differences in tissue-specific GLP-1 R expression, metabolic dependencies, and hormonal interactions. GLP-1 Rs are expressed in prostate, pancreatic, gastrointestinal, breast, gynecologic, and certain brain tumor cells, with increased expression reported in bile duct, esophageal, kidney, liver, ovarian, thyroid, and uterine cancers [40]. In contrast, lower expression has been observed in colon, lung, pancreatic, breast, head/neck, adrenal, and testicular tumors [40], highlighting marked heterogeneity across malignancies. Rather than representing a true inconsistency, this heterogeneity supports the notion that the potential anticancer effects of GLP-1 RAs may be mediated through multiple, partially independent mechanisms. While some effects may depend on receptor-independent pathways, including systemic metabolic improvements and sustained weight loss, GLP-1R functional activity influences downstream signaling pathways such as PI3K/Akt, AMPK, and Wnt/β-catenin, providing a mechanistic basis for the receptor-specific anticancer effects observed in preclinical studies. While epidemiological and clinical studies provide a general framework for understanding potential associations between GLP-1 RAs and cancer incidence, it is essential to examine these effects in the context of individual malignancies (Table 1).

**Table 1:** Summary of GLP1 RAs effects on specific cancer types

Cancer type	Current evidence
Prostate cancer	Lower incidence of prostate cancer in patients on GLP-1 RAs vs other anti-diabetic medications Inhibition of cancer cell proliferation through suppression of PI3K/Akt/mTOR and activation of AMPK signaling pathways Induction of apoptosis by increasing caspase-3 activity and downregulating anti-apoptotic proteins like BCL-2 Suppression of androgen receptor signaling Delay of tumor growth and reduction of metastasis by improving insulin sensitivity and reducing hyperinsulinemia and cancer-promoting signals like IGF-1
Breast cancer	Inhibition of cancer cell proliferation through suppression of PI3K/Akt/mTOR and activation of AMPK signaling pathways Reduction of tumor growth by downregulating cyclin D1 and upregulating pro-apoptotic markers like BAX Modulation of tumor microenvironment: decrease of inflammatory cytokines like TNF-α and IL-6
Pancreatic cancer	Reduction of tumor growth and inhibition of cancer cells proliferation through suppression of PI3K/Akt/mTOR and activation of AMPK signaling pathways Enhancement of immune response against cancer cells
Gastrointestinal and hepatic cancer	Lower incidence of colorectal cancer and hepatocellular carcinoma reported in observational studies of GLP-1 RA users vs other antidiabetic therapies Inhibition of cancer cell proliferation through suppression of PI3K/Akt, AMPK activation, and modulation of Wnt/β-catenin signaling Induction of apoptosis in colorectal and hepatic cancer cells Anti-inflammatory and immunomodulatory effects in gastrointestinal tissues Effects may be partly mediated by weight reduction, improved insulin sensitivity, and reduced hyperinsulinemia/IGF-1 signaling
Gynecologic cancer	Lower incidence of gynecologic cancer in Patients on GLP-1 RAs vs other anti-diabetic medications Modulation of tumor microenvironment: reduced expression of pro-tumorigenic cytokines Weight-dependent effect: improvement in metabolic parameters
Glioma	Inhibition of cancer cells proliferation and promotion of apoptosis through suppression of PI3K/Akt/mTOR and activation of AMPK signaling pathways Promotion of immune surveillance in tumor microenvironment enhancing NK cells
Oral squamous cell carcinoma	Reduction of inflammatory signaling (NF-κB pathways) Decrease of tumor cell viability Induction of apoptosis

*CRP* C-reactive protein, *GLP-1 RAs* GLP- 1 receptor agonists, *IGF-1* insulin-like growth factor 1; *IL-6* interleukin-6, *NK* natural killer, *TNF-α* tumor necrosis factor α

### Prostate cancer

Prostate cancer is among the most common cancers in men worldwide [41]. Recent studies have indicated that GLP-1 RAs may reduce prostate cancer risk and inhibit tumor progression. Epidemiological data suggest that patients on GLP-1 RA therapy exhibit a lower incidence of prostate cancer compared to those on other antidiabetic medications [42]. Mechanistically, GLP-1 RAs have been shown to inhibit prostate cancer cell proliferation by modulating signaling pathways such as PI3K/Akt and AMPK, which are key regulators of cell growth and metabolism [43].

In vitro studies demonstrate that liraglutide and semaglutide induce apoptosis in prostate cancer cell lines by increasing caspase-3 activity and downregulating anti-apoptotic proteins like Bcl-2 [44]. Additionally, GLP-1 RAs suppress androgen receptor signaling, which is critical for prostate cancer progression, suggesting their potential as adjunct therapies [45].

Furthermore, GLP-1 RAs improve insulin sensitivity and reduce hyperinsulinemia, indirectly lowering cancer-promoting signals such as insulin-like growth factor 1 (IGF-1), which is implicated in prostate cancer development [11, 12].

### Breast cancer

Preclinical studies have yielded heterogeneous results that appear highly context dependent. While exendin 4 and dulaglutide have shown inhibitory effects on breast cancer cell proliferation, liraglutide has displayed divergent actions across experimental models. In particular, proliferative effects have been reported in triple negative breast cancer models exposed to very high liraglutide concentrations, mediated by NOX4/ROS/VEGF signaling [47]. These findings contrast with other experimental studies showing inhibitory effects of liraglutide on breast cancer cell proliferation and migration at different doses and conditions [18].

Such preclinical observations have not been confirmed in clinical studies. Current evidence suggests that GLP-1 RAs do not increase the risk of breast cancer; in fact, they may be linked to a reduced risk of developing the disease [46]. Clinical data support the idea that GLP-1 RAs may have a direct antitumor effect on hormone receptor-positive breast cancer [47]. Additionally, in obese patients with T2D, weight loss induced by GLP-1 RAs has been associated with an increased detection rate of breast cancer, with the highest incidence occurring in those who lost more than 10% of their body weight during treatment [48, 49].

### Pancreatic cancer

Pancreatic ductal adenocarcinoma is characterized by poor prognosis and limited treatment options. Preclinical studies reveal that GLP-1 RAs can reduce pancreatic tumor growth and improve survival in animal models by inhibiting cancer cell proliferation and enhancing immune responses [50]. GLP-1 RAs activate AMPK and suppress PI3K/Akt/mTOR pathways, reducing cell survival and proliferation [51, 52].

### Gastrointestinal and hepatic cancers

Emerging evidence suggests that GLP-1 RAs may exert protective effects against gastrointestinal malignancies, including colorectal, gastric, and hepatocellular cancers. Preclinical studies demonstrate that GLP-1 RAs inhibit proliferation and induce apoptosis in colorectal cancer cell lines via modulation of PI3K/Akt, AMPK, and Wnt/β-catenin signaling pathways [32, 61]. In hepatocellular carcinoma (HCC) models, GLP-1 RAs reduce tumor growth and improve survival, likely through suppression of lipotoxicity, inflammation, and insulin/IGF-1–driven proliferative signals [32, 62].

Clinical and observational data support these preclinical findings. A meta-analysis of 90 studies found no excess risk for hepatic, biliary tract, pancreatic, colorectal, or gallbladder cancers in patients using GLP-1R therapies, whether prescribed for T2D or weight loss [53]. Other studies reported up to a 58% reduction in HCC risk in GLP-1 RA users compared with patients on insulin or other anti-hyperglycemic drugs [54, 55]. For colorectal cancer, pooled data from over 2 million individuals showed significantly lower risk in GLP-1 RA users versus TZDs, SGLT2 inhibitors, and insulin, while risk was comparable to sulfonylureas, metformin, or DPP4 inhibitors [56]. Interestingly, insulin therapy conferred a 69% higher risk, likely due to its mitogenic and prosurvival effects on colorectal cancer cells [56, 57].

Overall, these findings suggest that, in addition to weight reduction and metabolic improvements, GLP-1 RAs may provide direct antitumor effects in gastrointestinal tissues, potentially mediated by anti-inflammatory, immunomodulatory, and metabolic mechanisms.

### Endometrial, cervical and ovarian cancers

In gynecologic cancers, GLP-1 RAs show promise by modulating the tumor microenvironment. In vitro studies demonstrate decreased proliferation and increased apoptosis in endometrial and ovarian cancer cells exposed to GLP-1 RAs [23, 58]. These agents also reduce expression of pro-tumorigenic cytokines and chemokines, which are pivotal in tumor progression and metastasis [23, 58].

Early clinical evidence suggests that GLP-1 RA therapy in diabetic women correlates with lower incidence rates of endometrial and ovarian cancers, possibly through improved metabolic parameters such as insulin sensitivity and reduced adipose inflammation [2].

### Glioma

Gliomas are aggressive brain tumors with limited therapeutic options. Recent in vitro research shows that GLP-1 RAs inhibit glioma cell proliferation and promote apoptosis by activating AMPK and downregulating oncogenic pathways such as PI3K/Akt/mTOR [59]. Additionally, GLP-1 RAs may enhance NK cell activity, contributing to improved immune surveillance in the tumor microenvironment [30].

### Oral squamous cell carcinoma

GLP-1 RAs also exhibit anti-proliferative effects on oral squamous carcinoma cells [60]. Experimental data indicate that GLP-1 RAs reduce inflammatory signaling (e.g., NF-κB pathway), decrease tumor cell viability, and induce apoptosis. These effects suggest that GLP-1 RAs could be valuable adjuncts to current treatment regimens, especially in patients with metabolic comorbidities [60].

## Safety and limitations of current studies on GLP-1 receptor agonists in cancer prevention and treatment

**Fig. 2:**
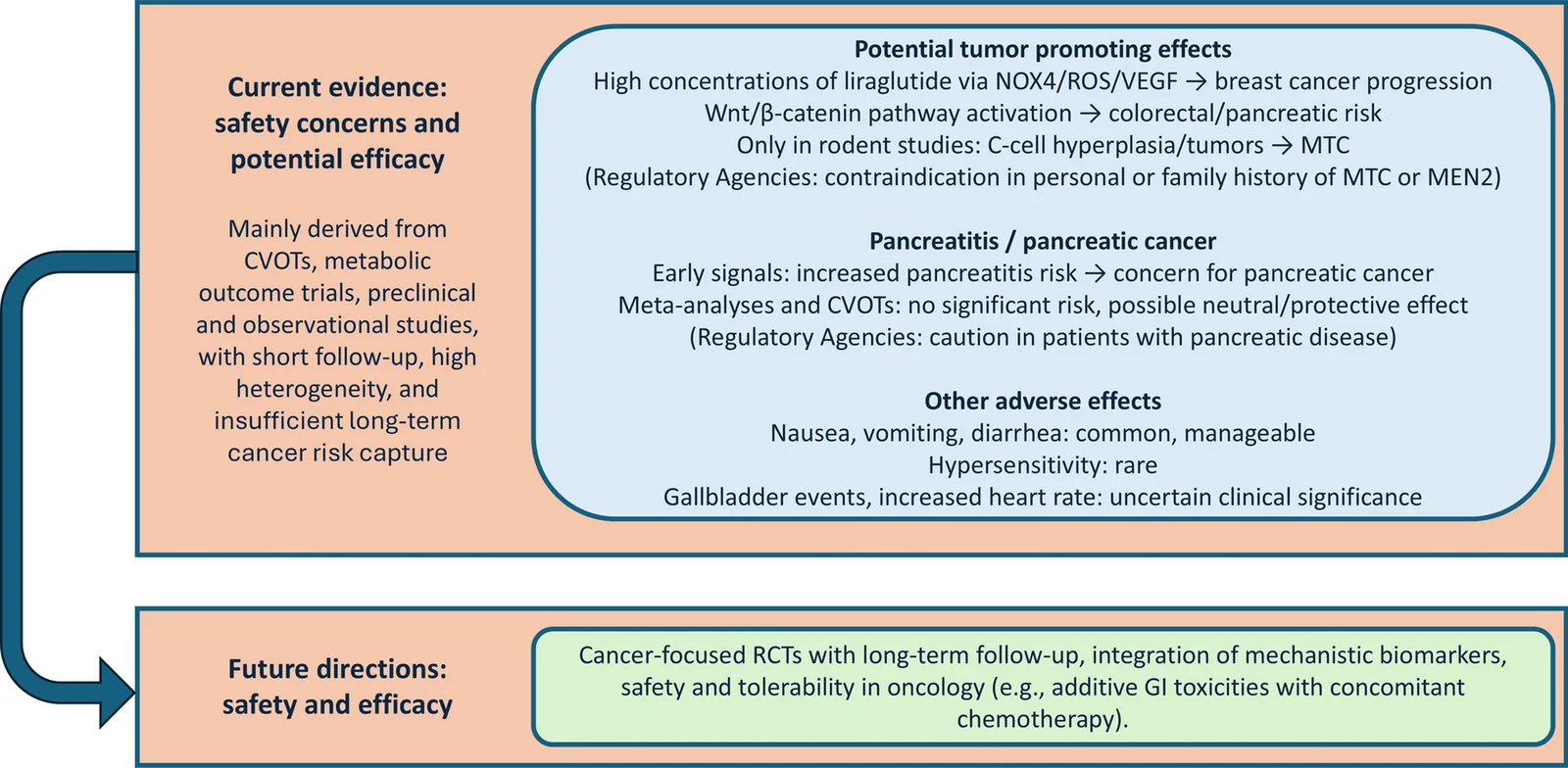
Safety concerns, current evidence limitation and future directions. *CVOTs* cardiovascular outcome trials, *GI* gastrointestinal, *MEN2* multiple endocrine neoplasia type 2, *MTC* medullary thyroid cell carcinoma, *RCTs* randomized clinical trials

Despite promising preclinical and observational data supporting the GLP-1 RAs role in cancer prevention and adjunctive treatment, there remain significant safety concerns and limitations in the current evidence base (Fig. 2). Evidence suggests potential for tumor promoting effects in certain contexts: high concentrations of liraglutide may promote progression of highly invasive breast cancer cell lines via NOX4/ROS/VEGF signaling, and theoretical concerns exist about Wnt/β catenin pathway activation potentially impacting colorectal and pancreatic carcinogenesis [61].

One of the most significant safety concerns raised pertains to the potential increased risk of MTC [62]. This concern originates primarily from rodent studies, where GLP-1 RAs, such as liraglutide, were shown to induce C-cell hyperplasia and tumors in the thyroid gland. However, these rodent findings have not been conclusively replicated in humans, possibly due to species-specific differences in GLP-1 R expression in thyroid C-cells. It should be noted that preclinical signals may have contributed to surveillance bias, as patients receiving GLP-1 RAs are often monitored more closely for thyroid and pancreatic malignancies than untreated individuals, potentially leading to earlier or more frequent detection. Moreover, confounding by indication should be considered, since underlying conditions such as obesity and T2D are themselves risk factors for cancer. For this reason, observational studies should be interpreted with caution. Nevertheless, large clinical trials and post-marketing surveillance have not demonstrated a statistically significant increase in MTC incidence among patients treated with GLP-1 RAs [62]. For example, the LEADER trial, which included over 9,000 patients treated with liraglutide, reported no cases of MTC attributable to the drug during a median follow-up of 3.8 years [63]. Similarly, other cardiovascular outcome trials (CVOTs) for GLP-1 RAs such as semaglutide and dulaglutide have not identified increased thyroid cancer risk [64, 65]. Moreover, in a meta‑analysis of 13 randomized controlled trials including 13,761 participants, although higher doses of tirzepatide were associated with greater increases in serum calcitonin levels, no cases of papillary thyroid carcinoma were reported in the included trials and tirzepatide exposure was not associated with an increased risk of overall or site-specific cancer compared with pooled control groups [66]. Nevertheless, regulatory agencies recommend caution, and GLP-1 RAs remain contraindicated in patients with a personal or family history of MTC or MEN2 [67].

Pancreatitis has been another area of concern linked to GLP-1 RA therapy [9]. Early post-marketing reports and observational studies suggested a possible increased risk of acute pancreatitis, which raised alarms about the safety of GLP-1 RAs, given the known association between pancreatitis and pancreatic cancer [68]. Some preclinical models indicated that GLP-1 RAs might influence pancreatic exocrine cells and inflammatory processes [69]. However, subsequent large-scale meta-analyses and CVOT data have not confirmed a significant elevation in pancreatitis risk. For example, a meta-analysis including over 50,000 participants found no statistically significant increase in pancreatitis incidence with GLP-1 RA use versus placebo or other therapies [70]. Moreover, no conclusive evidence links GLP-1 RA therapy to pancreatic cancer development; rather, some studies suggest a neutral or potentially protective effect [71]. Nonetheless, pancreatitis remains a labeled adverse effect in GLP-1 RA prescribing information, and clinicians are advised to monitor patients carefully, especially those with a history of pancreatic disease.

Beyond MTC and pancreatitis, other adverse effects have been documented with GLP-1 RAs, including gastrointestinal symptoms such as nausea, vomiting, and diarrhea [72]. These effects are common but generally transient and manageable. Rarely, severe hypersensitivity reactions have been reported [72].

Concerns have also been raised regarding gallbladder-related events and increased heart rate, but the clinical significance of these effects remains under investigation [73, 74]. Overall, the safety profile of GLP-1 RAs in metabolic disease is considered favorable, yet safety in oncology populations—who may have different vulnerabilities—requires more focused study.

## Challenges and future directions

The direct antitumor effects of GLP-1 RAs, independent of their weight-reducing capabilities, warrant further investigation in the context of cancer prevention and active treatment. Given their safety profile and widespread clinical use for metabolic diseases, repurposing GLP-1 RAs in oncology could provide a novel adjunct to existing therapies. However, several challenges remain that future research must address. The precise molecular targets of GLP-1 RAs in different tumor types require further elucidation. Moreover, heterogeneity in GLP-1 receptor expression across cancers may influence therapeutic efficacy. Biomarker development will be critical to identify patients most likely to benefit from GLP-1 RA–based therapies. Furthermore, it is necessary to explore potential synergistic effects of GLP-1 RAs with immune checkpoint inhibitors and targeted therapies, aiming to maximize anti-tumor efficacy. Understanding the immunomodulatory impact on the tumor microenvironment will also be a key research topic. An additional and emerging area of interest concerns the reported effects of GLP-1 RAs on reward-related behaviors and hedonic substance use [75]. Growing evidence suggests that GLP-1 signaling within central nervous system reward pathways may reduce alcohol intake, smoking behavior, and other addictive patterns [76, 77]. Considering the well-established role of alcohol consumption and tobacco use as major modifiable cancer risk factors, it is conceivable that part of the observed cancer risk reduction associated with GLP-1 RA therapy may be indirectly mediated through favorable changes in these behaviors. Although current evidence remains preliminary and largely derived from preclinical studies and observational human data, this neurobehavioral dimension warrants further investigation in prospective studies specifically designed to assess lifestyle modifications as potential mediators of cancer-related outcomes.

Despite the promising signals from preclinical and observational studies, several critical limitations must be acknowledged to ensure a balanced interpretation of the role of GLP-1 RAs in obesity-related cancers. Current evidence is primarily derived from secondary analyses of trials not originally designed for oncological endpoints and observational cohorts, which are susceptible to selection bias and confounding by indication. Furthermore, the significant heterogeneity in GLP-1 RA formulations, dosages, and treatment durations—combined with context-dependent preclinical findings, such as potential pro-proliferative effects in specific breast cancer models—precludes definitive conclusions. Also, existing trials are often too short to capture long-term cancer risk (Fig. 2).

An additional limitation of the current evidence is the lack of consistent sex-stratified analyses, excluding sex-specific malignancies. Most large trials and observational studies report pooled outcomes, limiting the ability to assess whether the anticancer effects of GLP-1 RAs differ between men and women. Biological differences in adipose tissue distribution, hormonal milieu, insulin sensitivity, and immune responses may influence both baseline cancer risk and response to GLP-1 RA therapy. Moreover, sex-related variability in tumor microenvironment composition and inflammatory signaling could potentially modulate treatment effects.

Future research should prioritize cancer-focused randomized controlled trials with clearly defined design features. Target populations may include individuals with obesity (with or without T2D) at high risk for obesity-related cancers, as well as selected patients with early-stage malignancies in the adjuvant setting. Prevention trials will require prolonged follow-up (ideally ≥ 5 years) to adequately capture incident cancer outcomes. Primary endpoints should include site-specific cancer incidence, cancer-related mortality, or progression-free survival, depending on the clinical context, together with rigorous safety assessment. Appropriate comparator arms (e.g., placebo, standard metabolic therapies, or bariatric strategies) and prespecified stratification by sex and metabolic profile will be essential to clarify causal effects and identify patient subgroups most likely to benefit.

Ethical and feasibility considerations should also be contextualized within current regulatory indications. GLP-1 RAs are already approved for the treatment of obesity and T2D, but their use in overweight non-diabetic oncologic individuals would require careful risk–benefit evaluation and robust clinical evidence before routine implementation could be justified. Furthermore, issues related to cost, long-term adherence, and equitable access should be considered when proposing preventive strategies.

In conclusion, future research must transition toward randomized controlled trials with standardized oncological biomarkers and long-term safety monitoring. Until then, the clinical use of these agents in patients with complex oncological profiles should remain cautious and highly individualized, considering potential interactions with standard antineoplastic therapies.

## Conclusions

GLP-1 RAs represent a class of agents with established metabolic benefits and a potential role in cancer biology that remains to be further elucidated. Their modulation of insulin sensitivity, inflammatory pathways, and cellular proliferation supports a plausible role in cancer prevention and adjunctive therapy. Nonetheless, current evidence is insufficient to definitively confirm their safety and efficacy in oncology settings.

Although rodent studies raised concerns regarding MTC, human data have not substantiated a significant risk. Similarly, initial signals of pancreatitis and pancreatic cancer risk have been largely attenuated by subsequent rigorous analyses. Common side effects, such as gastrointestinal discomfort, are usually manageable and transient, but rare adverse events warrant vigilance, particularly in vulnerable patient populations.

The absence of dedicated large-scale randomized controlled trials with cancer-specific endpoints remains a critical limitation. Existing data largely comes from secondary analyses or observational cohorts with inherent biases and short follow-up durations, restricting the ability to assess long-term oncologic outcomes.

Future research must prioritize well-designed, adequately powered cancer-focused RCTs that evaluate both efficacy and safety over extended periods. Integration of biomarker studies and mechanistic investigations will also be essential to understand the molecular effects of GLP-1 RAs on tumor biology. Until such evidence emerges, clinicians should carefully weigh potential benefits against unresolved safety concerns, tailoring decisions to individual patient risk profiles.

In summary, GLP-1 RAs are characterized by established metabolic benefits and emerging anticancer signals, but any translation into oncology should proceed cautiously and be supported by robust clinical evidence.

## Data Availability

Not applicable.
